# Commensal Bacteria Impact on Intestinal Toll-like Receptor Signaling in *Salmonella*-Challenged Gnotobiotic Piglets

**DOI:** 10.3390/pathogens12111293

**Published:** 2023-10-29

**Authors:** Alla Splichalova, Zdislava Kindlova, Jiri Killer, Vera Neuzil Bunesova, Eva Vlkova, Barbora Valaskova, Radko Pechar, Katerina Polakova, Igor Splichal

**Affiliations:** 1 Laboratory of Gnotobiology, Institute of Microbiology, Czech Academy of Sciences, 549 22 Novy Hradek, Czech Republic; splichalova@gnotobio.cz (A.S.); kindlova@gnotobio.cz (Z.K.); valaskova@gnotobio.cz (B.V.); polakova@gnotobio.cz (K.P.); 2 Department of Microbiology, Nutrition and Dietetics, Faculty of Agrobiology, Food and Natural Resources, Czech University of Life Sciences Prague, 165 00 Prague, Czech Republic; killer@iapg.cas.cz (J.K.); bunesova@af.czu.cz (V.N.B.); vlkova@af.czu.cz (E.V.); pecharr@af.czu.cz (R.P.); 3 Institute of Animal Physiology and Genetics, Czech Academy of Sciences, 142 20 Prague, Czech Republic; 4 Department of Research, Food Research Institute Prague, 102 00 Prague, Czech Republic

**Keywords:** *Bifidobacterium*, cytokines, gnotobiotic minipig, *Lactobacillus*, lipopolysaccharide, *Salmonella* Typhimurium, Toll-like receptor

## Abstract

Gnotobiotic (GN) animals with simple and defined microbiota can help to elucidate host-pathogen interferences. Hysterectomy-derived germ-free (GF) minipigs were associated at 4 and 24 h post-hysterectomy with porcine commensal mucinolytic *Bifidobacterium boum* RP36 (RP36) strain or non-mucinolytic strain RP37 (RP37) or at 4 h post-hysterectomy with *Lactobacillus amylovorus* (LA). One-week-old GN minipigs were infected with *Salmonella* Typhimurium LT2 strain (LT2). We monitored histological changes in the ileum, mRNA expression of Toll-like receptors (TLRs) 2, 4, and 9 and their related molecules lipopolysaccharide-binding protein (LBP), coreceptors MD-2 and CD14, adaptor proteins MyD88 and TRIF, and receptor for advanced glycation end products (RAGE) in the ileum and colon. LT2 significantly induced expression of TLR2, TLR4, MyD88, LBP, MD-2, and CD14 in the ileum and TLR4, MyD88, TRIF, LBP, and CD14 in the colon. The LT2 infection also significantly increased plasmatic levels of inflammatory markers interleukin (IL)-6 and IL-12/23p40. The previous colonization with RP37 alleviated damage of the ileum caused by the *Salmonella* infection, and RP37 and LA downregulated plasmatic levels of IL-6. A defined oligo-microbiota composed of bacterial species with selected properties should probably be more effective in downregulating inflammatory response than single bacteria.

## 1. Introduction

### 1.1. Pathogen- and Damage-Associated Molecular Patterns

Inflammation is a protective reaction to keep homeostasis and organ integrity [1]. The inflammatory process is triggered by recognizing molecular patterns via pattern recognition receptors (PRRs) [2]. These molecular patterns are divided into pathogen-associated molecular patterns (PAMPs) and damage-associated molecular patterns (DAMPs) [3,4]. PAMPs are foreign (extrinsic) molecular structures typical for microorganisms [3], but DAMPs are host (intrinsic) body structures [5,6]. DAMPs are generally hidden from immune recognition but become available after their release by tissue damage or by secretion [4,6].

Toll-like receptors (TLRs) are one group of PRRs that sense PAMPs and DAMPs. They recognize PAMPs as hetero- or homodimers, e.g., bacterial PAMPs—TLR2/TLR1 (triacyl lipopeptide), TLR2/6 (diacyl lipopeptide), TLR4/TLR4 (lipopolysaccharide), TLR5/TLR5 (flagellin), and TLR9/TLR9 (CpG motif) [7,8]. However, the repertoire of each TLR is not limited to one PAMP but usually covers a broader panel of PAMPs [8,9]. Moreover, TLRs also recognize DAMPs, e.g., uric acid, the myosin heavy chain, SAP130 and S100 proteins, ATP, nucleic acids including mitochondrial DNA, and intracellular nuclear DNA-binding protein high mobility group box 1 (HMGB1) [10]. HMGB1 extracellular presence is sensed with a receptor for advanced glycation end (RAGE), TLR2, TLR4, TLR9, and other receptors [11]. This TLR overlapping [12] explains the similarity in consequences of sepsis and sterile inflammation [13] that are triggered by the recognition of PAMPs or DAMPs, respectively [4,9,14].

Lipopolysaccharide (LPS) is a Gram-negative bacteria cell wall component released from bacterial cells after their death and destruction [15,16]. It is a central molecule of Gram-negative bacteria-caused sepsis [17], triggering the unregulated release of inflammatory mediators called the “cytokine storm” that provokes multiorgan failure, often resulting in the host’s death [18]. TLR4 is an essential TLR for LPS [7]. The released LPS is initially trapped and cumulated by lipopolysaccharide-binding protein (LBP) and presented to the CD14 molecule [15,16]. CD14 transports LPS to the TLR4/MD-2 complex, which recognizes it and triggers the downstream cascade and production of inflammatory cytokines [1]. Cytokines participate at their physiological levels in homeostasis [19], but their exaggerated levels harm the organism [1].

### 1.2. Colonization of the Newborn Intestinal Tract and Salmonella Typhimurium

Newborn infants are settled with primary microbiota dependent on their delivery [20]. *Lactobacillus* spp. predominate in the vaginal microbiome. Thus, lactobacilli belong among the first colonizers of the newborn intestine in vaginally-born infants [21]. They co-form conditions, e.g., consume oxygen and reduce pH, that are suitable for settlement of their followers, e.g., obligatory anaerobic *Bifidobacterium* spp. that are the principal inhabitants of an infant’s intestine [20,22]. The stable colonization, the creation of a balanced microbiota, and its persistence in the intestine are prerequisites for the beneficial effects of commensal bacteria on host health [23]. Both bifidobacteria and lactobacilli are the main components of multi-strain probiotic preparations [23,24,25]. On the contrary, dysbiosis contributes to decreased host colonization resistance and facilitates diarrheagenic diseases [26,27].

Around 40% of all diarrheal disease-associated deaths are attributed to *Salmonella* [28]. The genus *Salmonellae* consists of around 2500 serovars that are human and animal orally acquired pathogens [29,30]. *Salmonella enterica* (*S. enterica*) serovars can cause, in dependence on a host and *Salmonella* serovar, four major syndromes: enteric fever (typhoid), enterocolitis/diarrhea, bacteremia, and chronic asymptomatic carriage [31]. The serovar Typhimurium (*S*. Typhimurium) causes self-limiting enterocolitis in formerly healthy immunocompetent individuals [32]. However, in risk subjects, e.g., children < 1 year of age and HIV-infected persons, the infection can cause life-threatening disease with more severe progress [33,34].

### 1.3. Pig Translational Model

The pig shows close similarities to humans in genetics, physiology, anatomy, and microbiome [35,36,37]. This predetermines pigs as animal models in many areas of biomedical research, including gastroenterology [38,39], immunology [40,41], and infectious diseases [42]. Moreover, the pig has epitheliochorial placentation [43] that prevents the prenatal transfer of protective immunoglobulins from the mother to the fetus, unlike in humans [44]. A newborn piglet obtains protective immunoglobulins and immunocytes through colostrum intake after birth [45]. The fact that mammalian fetuses develop in sterile conditions of the uterus [46,47,48] allows deriving sterile (germ-free; GF) animals [49,50] for the study of microbiota-host interactions in microbiologically-controlled (gnotobiotic; GN) conditions [47,51]. Surgically derived GF piglets show less colonization resistance than conventional (CV) ones [52,53]. This results in the higher sensitivity of the GF animals to enteric infections [54]. Therefore, GF piglets are used for in vivo studies of host and microbiota relationships, including the importance of bacterial virulence factors [52,53].

This work aimed to describe how pig commensal bacteria—mucinolytic *Bifidobacterium boum* RP36, non-mucinolytic *Bifidobacterium boum* RP37, and *Lactobacillus amylovorus* DSM 16698^T^ interfere with *Salmonella* Typhimurium LT2 in GN piglets. The modulatory properties of the bacteria were evaluated using TLR2, TLR4, TLR9, and RAGE mRNA expression, leading to the release of inflammatory cytokines IL-6 and IL-12/23p40.

## 2. Materials and Methods

### 2.1. Gnotobiotic Minipigs

Germ-free (GF) minipigs (Animal Research Institute, Kostelec nad Orlici, Czechia) were derived through hysterectomy on the 112th day of gestation, a full term for these pigs. The piglets were reared in fiberglass isolators with a heated floor and fed through a nipple with cow’s milk-based formula 6–7 times per day, and their microbiological state was tested as described elsewhere [55].

### 2.2. Bacterial Cultures

Pig commensal mucinolytic *B. boum* strain RP36 (*B. boum* RP36 or RP36), non-mucinolytic *B. boum* strain RP37 (*B. boum* RP37 or RP37) [56,57], *Lactobacillus amylovorus* DSM 16698^T^ (LA) [58], and *Salmonella* Typhimurium LT2 (*S*. Typhimurium or LT2) [59,60] were used in the present experiments. *B. boum* strains, *L. amylovorus*, and *S*. Typhimurium were cultivated as previously described [57,61].

### 2.3. Experimental Design

**Figure 1:**
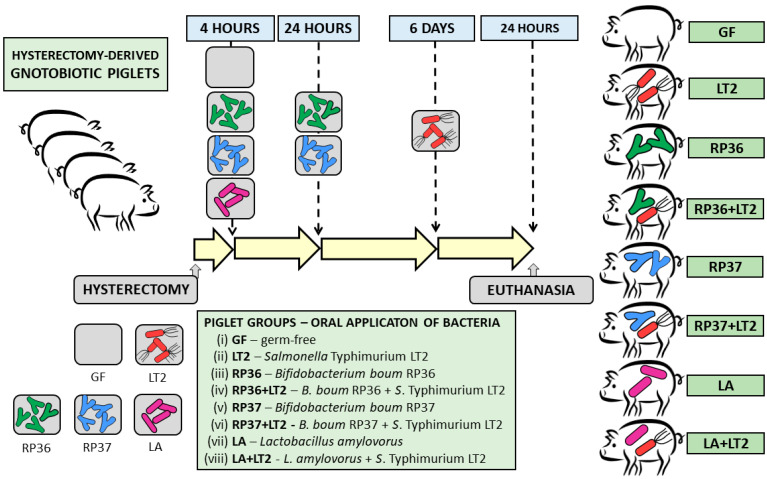
Schema of the experiment. The GN minipigs (n = 48) were divided into eight groups with six piglets per group: (i) GF; (ii) infected with LT2 (LT2); (iii) associated with RP36 (RP36); (iv) RP36 challenged with LT2 (RP36+LT2); (v) associated with RP37 (RP37); (vi) RP37 challenged with LT2 (RP37+LT2); (vii) associated with LA (LA); (viii) LA challenged with LT2 (LA+LT2).

Forty-eight GN minipigs from three independent hysterectomies were divided into eight groups containing six minipigs per group (Figure 1): (i) GF during the whole experiment (GF); (ii) challenged with 6 log *S.* Typhimurium LT2 CFU (LT2); (iii) repeatedly associated with 8 log *B. boum* RP36 CFU (RP36); (iv) RP36 challenged with 6 log LT2 CFU (RP36+LT2); (v) repeatedly associated with 8 log *B. boum* RP37 CFU (RP37); (vi) RP37 challenged with 6 log LT2 CFU (RP37+LT2); (vii) associated with 8 log *L. amylovorus* CFU (LA); and (viii) LA challenged with 6 log LT2 CFU (LA+LT2). Bacterial suspensions were applied per os in 5 mL of milk diet; GF piglets obtained the milk only. The groups LT2, RP36+LT2, RP37+LT2, and LA+LT2 were challenged with LT2 for 24 h. The isoflurane-anesthetized GN minipigs were exsanguinated via cardiac puncture, and required tissue samples were collected.

### 2.4. Clinical Signs

The minipigs were examined for signs of enterocolitis (fever, anorexia, sleepiness, and diarrhea) at each feeding by staff.

### 2.5. Histological Evaluation

The terminal ileum was fixed in Carnoy’s fluid for 30 min, dehydrated, and embedded in paraffin. Hematoxylin-eosin stained 5 μm cross-sections were examined under an Olympus BX 40 microscope with an Olympus Camedia C-2000 digital camera (Olympus, Tokyo, Japan). Ten measurements for each parameter were taken per minipig to appraise ileal villus length and crypt depth. Histological scoring was evaluated as previously described [61]: (i) submucosal edema (0–2); (ii) infiltration of polymorphonuclear neutrophils into the lamina propria (0–2); (iii) villus atrophy (0–3); (iv) exudate in lumen (0–2); (v) vessel dilatation (0–2); (vi) inflammatory cellularity in lymphatic vessel lumen (0–2); (vii) hyperemia (0–2); (viii) peritonitis (0–1), and (ix) erosion of the epithelial layer (0–3). The total score could reach 0–19 points [62].

### 2.6. Blood Plasma

Blood plasma was obtained via centrifugation from cooled citrated blood [57]. Protease inhibitors (Roche Diagnostics, Manheim, Germany) were added to plasma samples and then stored at −45 °C until following the processing.

### 2.7. Total RNA Extraction and cDNA Synthesis

Cross-sections of the terminal ileum and transverse colon were cut and stored in RNAlater (Sigma-Aldrich, St. Louis, MO, USA) at −20 °C until further homogenization with 2 mm zirconia beads (BioSpec Products, Bartlesville, OK, USA) in TissueLyser LT beadbeater (Qiagen, Hilden, Germany) and extraction with an RNeasy Mini Kit Plus (Qiagen). cDNA synthesis was performed from 500 ng of total RNA with a QuantiTect Reverse Transcription kit (Qiagen), [55]. Eighty μL of PCR quality water (Life Technologies, Carlsbad, CA, USA) was added to the synthesized cDNA. These cDNA templates were stored at −25 °C until Real-Time PCR.

### 2.8. Real-Time PCR

Two μL of the cDNA template was used to perform lock nucleic acid (LNA) probe (Universal ProbeLibrary; Roche Diagnostics)-based Real-Time PCR with β-actin and cyclophilin A as reference genes. The PCR amplification was performed on an iQ cycler (Bio-Rad, Hercules, CA, USA), and relative TLR4, MD-2, CD14, LBP, MyD88, TRIF, TLR2, TLR9, and RAGE mRNA expressions (fold changes) were counted using GenEx 6 software (MultiD Analyses AB, Gothenburg, Sweden) [55].

### 2.9. IL-6 and IL-12/23p40 in Blood Plasma

A Porcine ProcartaPlex kit (Affymetrix, Santa Clara, CA, USA) based on paramagnetic beads Luminex xMAP technology (Luminex Corporation, Austin, TX, USA) was used to measure IL-12/23p40 and IL-6 in plasma on a Bio-Plex Multi Array System with Bio-Plex Manager 4.01 software (Bio-Rad, Hercules, TX, USA) and evaluated as previously described [61].

### 2.10. Statistical Analysis

Values of all groups were checked for normality of their distribution and outliers. A one-way analysis of variance (ANOVA) with Tukey’s multiple comparisons post-hoc test was used for the statistical comparisons at *p* < 0.05 and evaluated with GraphPad Prism 6 software (GraphPad Software, San Diego, CA, USA). A letter system was used to depict the significance of group differences in graphs.

## 3. Results

### 3.1. Clinical Signs of Infection

All non-infected minipigs thrived for the whole experiment or until the challenge with *S*. Typhimurium. In contrast, clinical signs of enteric infection, such as fever, anorexia, sleepiness, and diarrhea, were observed in all infected minipigs (LT2, RP36+LT2, RP37+LT2, and LA+LT2) beginning 2–4 h after infection. Previous association with RP37 mildly delayed the manifestation of the infection. This was not observed in the previous association with RP36 and LA.

### 3.2. Histological Assessment of the Terminal Ileum

**Figure 2:**
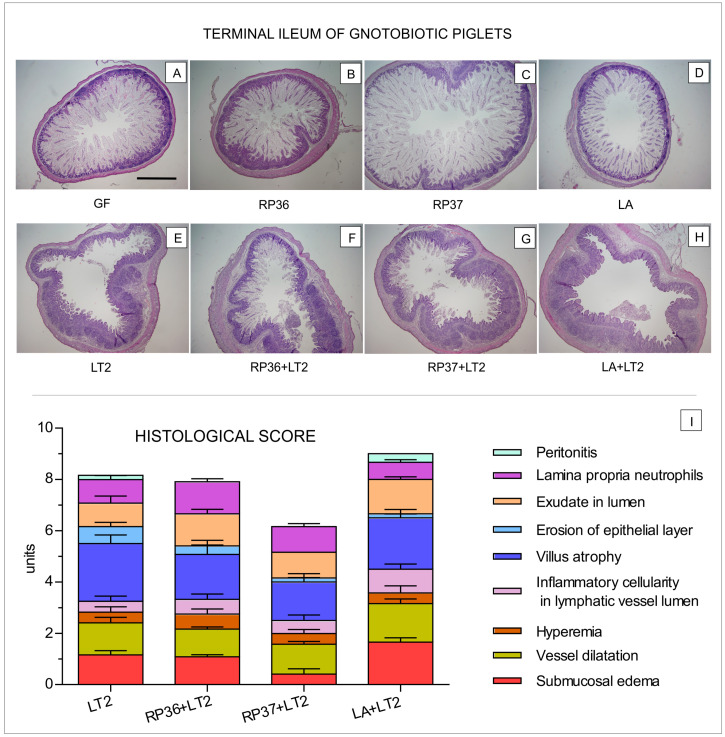
Representative hematoxylin-eosin stained terminal ileum cross sections in the GN minipigs and a histological score. The GN minipigs were divided into eight groups with six minipigs per group: (i) GF ((**A**), GF); (ii) associated with RP36 ((**B**), RP36); (iii) associated with RP37 ((**C**), RP37); (iv) associated with LA ((**D**), LA); (v) infected with LT2 ((**E**), LT2); RP36 challenged with LT2 ((**F**), RP36+LT2); RP37 challenged with LT2 ((**G**), RP37+LT2); and LA challenged with LT2 ((**H**), LA+LT2). The depicted bar on the cross-section (**A**) represents 1 mm. Histological score of six minipigs of four *Salmonella*-challenged minipig groups (LT2, RP36+LT2, RP37+LT2, and LA+LT2) are shown (**I**).

The evaluation of hematoxylin-eosin stained ileum of non-infected GF, RP36, RP37, and LA minipigs was performed only because villi of the infected LT2, RP36+LT2, RP37+LT2, and LA+LT2 minipigs were damaged and did not allow evaluation (Figure 2). No statistical differences (*p* < 0.05) in the villus height, crypt depth, and ratio of villus height/crypt depth were found among groups of non-infected GN minipigs (Table 1).

The ileum of the non-infected minipigs (GF, RP36, RP37, and LA) contained villi with vacuolated enterocytes that were located along the whole length of the villus from the top of crypts to the villus tips (Figure 2A–D). Because no obvious inflammatory signs or damage were observed in these minipigs, they are not included in the histological assessment depicted as the histological score graph (Figure 2I). In contrast, the ileum in the *Salmonella*-infected minipigs (LT2, RP36+LT2, RP37+LT2, and LA+LT2; Figure 2E–H) showed signs of acute inflammation and are summarized on the histological score graph (Figure 2I). The total histological score of the *Salmonella*-infected minipigs ranged between six and nine, with the lowest value in the RP37+LT2 and the highest in the LA+LT2 minipigs.

### 3.3. Toll-like Receptors, Their Related Molecules, and RAGE mRNA Expression in the Ileum

**Figure 3:**
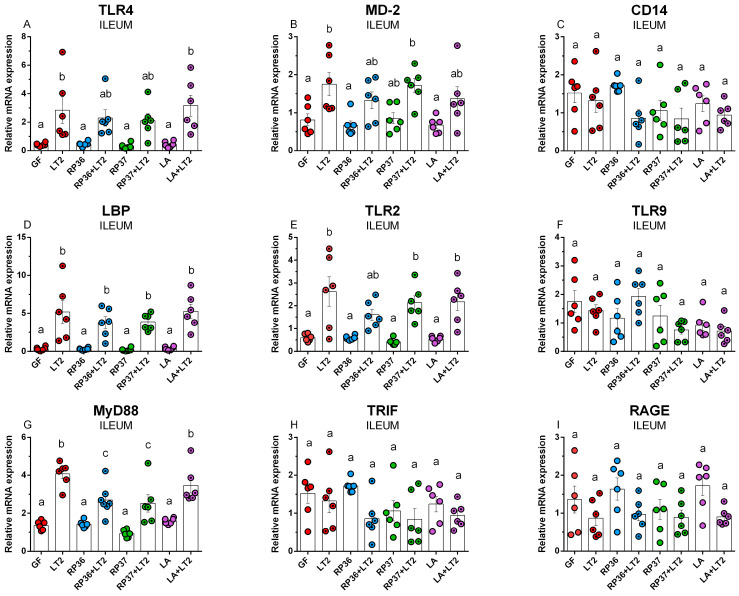
Expression of TLR4 (**A**), MD-2 (**B**), CD14 (**C**), LBP (**D**), TLR2 (**E**), TLR9 (**F**), MyD88 (**G**), TRIF (**H**), and RAGE (**I**) in the ileum of the GN minipigs: (i) GF, (ii) infected with LT2 (LT2), (iii) associated with RP36 (RP36), (iv) associated with RP36 and challenged with LT2 (RP36+LT2), (v) associated with RP37 (RP37), (vi) associated with RP37 and challenged with LT2 (RP37+LT2), (vii) associated with LA (LA), and (viii) associated with LA and challenged with LT2 (LA+LT2). The values are presented as individual dots indicating mean ± SEM. Statistical differences were evaluated using one-way ANOVA with Tukey’s post-hoc test, and *p* < 0.05 is denoted with different letters above the columns. Six samples in each group were analyzed.

TLR4 mRNA expression was induced in the LT2-infected minipig groups (LT2, RP36+LT2, RP37+LT2, and LA+LT2) compared to *Salmonella*-free (GF, RP36, RP37, and LA) groups (Figure 3A), but this increase was significant in the LT2 and LA+LT2 groups only. The preliminary one-week association with *B. boum* strains RP36 or RP37 prevented significant induction of TLR4 mRNA in the ileum of the RP36+LT2 and RP37+LT2 minipig groups. In contrast to both *B. boum* strains, *L. amylovorus* (LA+LT2) did not show this effect. The infection with LT2 significantly upregulated MD-2 mRNA expression (Figure 2B). The previous associations with RP36 (RP36+LT2) and LA (LA+LT2) downregulated MD-2 expression, but this downregulation was insignificant. The infection with *Salmonella* downregulated the mRNA expression of TLR4 coreceptor CD14 (Figure 3C). However, this downregulation was not statistically significant. LBP mRNA expression was induced in all cases of the *Salmonella*-infected minipigs (Figure 3D). Neither *B. boum* nor *L. amylovorus* ameliorated this induction. TLR2 mRNA expression was induced in the *Salmonella* infection, but previous association with mucinolytic *B. boum* RP36 prevented statistically significant induction of TLR2 mRNA (Figure 3E). In contrast, no influence of commensal bacteria on mRNA expression was shown in TLR9 (Figure 3F). The infection with *Salmonella* statistically significantly induced adaptor protein MyD88 mRNA, but this induction was significantly lower for previous association with either RP36 or RP37 *B. boum* strains (Figure 3G). Non-infected minipigs versus their infected counterparts (GF vs. LT2, RP36 vs. RP36+LT2, RP37 vs. RP37+LT2, and LA vs. LA+LT2) showed increased expression of TRIF mRNA (Figure 3H) and RAGE mRNA (Figure 3I) after infection with *Salmonella*. However, neither in the case of TRIF nor RAGE was this lowering statistically significant.

### 3.4. Toll-like Receptors, Their Related Molecules, and RAGE mRNA Expression in the Colon

**Figure 4:**
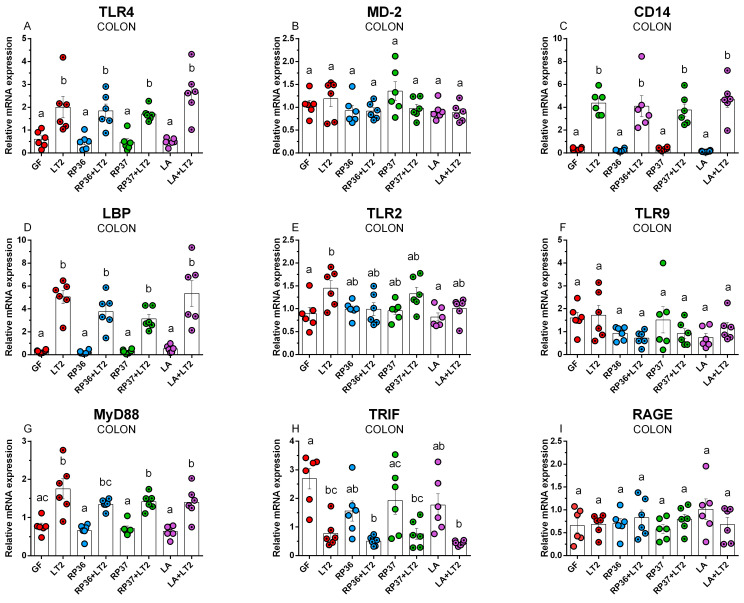
Expression of TLR4 (**A**), MD-2 (**B**), CD14 (**C**), LBP (**D**), TLR2 (**E**), TLR9 (**F**), MyD88 (**G**), TRIF (**H**), and RAGE (**I**) in the colon of the GN minipigs: (i) GF, (ii) infected with LT2 (LT2), (iii) associated with RP36 (RP36), (iv) associated with RP36 and challenged with LT2 (RP36+LT2), (v) associated with RP37 (RP37), (vi) associated with RP37 and challenged with LT2 (RP37+LT2), (vii) associated with LA (LA), and (viii) associated with LA and challenged with LT2 (LA+LT2). The values are presented as individual dots indicating mean ± SEM. Statistical differences were evaluated using one-way ANOVA with Tukey’s post-hoc test, and *p* < 0.05 is denoted with different letters above the columns. Six samples in each group were analyzed.

The infection with *Salmonella* upregulated statistically significantly TLR4 mRNA expression in all *Salmonella*-challenged minipigs (LT2, RP36+LT2, RP37+LT2, and LA+LT2) compared to non-challenged ones (GF, RP36, RP37, and LA) (Figure 4A). No statistically significant differences among infected and non-infected minipig groups were found. In contrast, no differences in MD-2 expression were found among all minipig groups (Figure 4B). TLR4 coreceptor CD14 mRNA expression faithfully copied TLR4 mRNA expression and showed upregulated expression after infection with *Salmonella* without any influence of previous association with commensal bacteria (Figure 4C). A concordant infection-stimulated upregulation without the influence of previous association was shown also in LBP mRNA expression (Figure 4D). TLR2 mRNA was statistically significantly upregulated in *Salmonella*-infected LT2 minipigs compared to the GF group (Figure 4E). Previous association with RP36, RP37, or LA ameliorated this upregulation, and these infected groups did not statistically differ from either the GF or LT2 groups. The association with both *B. boum* strains resulted in nonsignificant differences among them, the GF, and the LT2 groups. Association with only LA did not influence the TLR2 mRNA expression comparable to the GF one. Neither association nor infection significantly influenced the expression of TLR9 mRNA, which was comparable in all groups (Figure 4F). The infection with *Salmonella* upregulated MyD88 mRNA expression. However, significant differences were found among the infected LT2, RP37+LT2, and LA+LT2 groups and non-infected RP36, RP37, and LA only groups (Figure 4G). TRIF mRNA showed a downregulated trend after infection with *Salmonella,* and significant downregulation was shown in all *Salmonella*-infected minipig groups compared to the GF group (Figure 4H). RAGE mRNA expression did not show significant regulation influenced by association with commensal bacteria or infection with *Salmonella* (Figure 4I).

### 3.5. IL-6 and IL-12/23p40 Levels in Plasma

**Figure 5:**
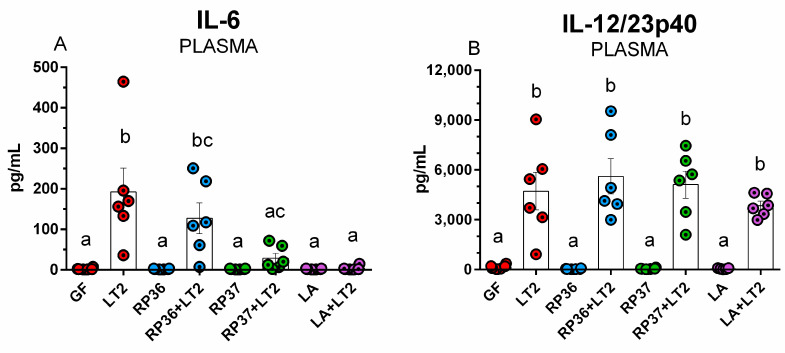
Plasmatic levels of IL-6 (**A**) and IL-12/23p40 (**B**) in the GN minipigs: (i) GF, (ii) infected with LT2 (LT2), (iii) associated with RP36 (RP36), (iv) associated with RP36 and challenged with LT2 (RP36+LT2), (v) associated with RP37 (RP37), (vi) associated with RP37 and challenged with LT2 (RP37+LT2), (vii) associated with LA (LA), and (viii) associated with LA and challenged with LT2 (LA+LT2). The values are presented as individual dots indicating mean ± SEM. Statistical differences were evaluated using one-way ANOVA with Tukey’s post-hoc test, and *p* < 0.05 is denoted with different letters above the columns. Six samples in each group were analyzed.

The GF minipigs colonized with pig commensal bacteria (RP36, RP37, and LA) showed comparable levels of IL-6 (Figure 5A). The infection with *S*. Typhimurium significantly increased plasmatic IL-6 levels. The previous association with RP37 (RP37+LT2) and LA (LA+LT2) significantly downregulated IL-6 levels compared to the LT2 group. In the case of IL12/23p40 (Figure 5B), the infection with *S*. Typhimurium also, as in the case of IL-6, significantly increased plasmatic IL-12/23p40 levels. The previous association with pig commensal bacteria (RP36, RP37, and LA) did not influence this increase.

## 4. Discussion

Both lactobacilli and bifidobacteria are the most common components of single- or multi-strain probiotic preparations [23,24,25]. TLR2 is recognized as a pivotal TLR for peptidoglycan, lipopeptides, and lipoproteins of Gram-positive bacteria, mycoplasma lipopeptides, or fungal zymosan and β-glucan. The primary TLR4 ligand of Gram-negative bacteria is lipopolysaccharide (LPS) [7]. However, the possible strict recognition of Gram-positive and Gram-negative bacteria by TLR2 and TLR4, respectively, is more complicated, as we discuss later.

*S.* Typhimurium-caused enterocolitis is most severe in the terminal ileum and proximal colon, and neutrophil recruitment to the intestinal epithelium is the hallmark of this enteric disease [31,63]. Hematoxylin-eosin staining of terminal ileum cross sections showed that the absence of bacteria in the GF minipigs or association with pig commensal bacteria in the RP36, RP37, and LA groups did not influence intestinal histology, and villi contained vacuolated enterocytes that were typical for newborn piglets [64,65]. The association with microbiota stimulated the disappearance of vacuoles [66]. The terminal ileum is the main site of *Salmonella* detrimental attack and translocation [67,68]. Thus, we targeted our attention to the histological structure of the ileum.

The infection with *Salmonella* roughly disrupted intestinal architecture; the villi containing vacuolated enterocytes were shortened, and most vacuoles disappeared. A degree of damage was assessed with the histological scoring established for preterm [62] and term GN minipigs [61]. The previous association with these pig commensal bacteria did not prevent the damage of the mucosa and is comparable with the association of GN minipigs with our formerly isolated pig commensal *Lactobacillus mucosae* P5 and *L. amylovorus* P1 [61]. Any intestinal barrier damage facilitates translocation [67]. The mono-association with all these pig-derived bifidobacteria and lactobacilli showed no or lower protective effect against intestinal damage than association with probiotic bacteria *Escherichia coli* Nissle 1917 (EcN) [69], which showed a substantial protective effect [61].

We attempted to regulate TLR4/MD-2 signaling and the consequent production of inflammatory cytokines (here represented by IL-6 and IL-12/23p40) in GN minipigs using pig commensal bacteria *B. boum* strains RP36 and RP37 [57] and LA [70]. Our attention was focused on indigenous *Bifidobacterium* and *Lactobacillus* spp. as essential species of primary microbiota [20,21].

The expressions of TLR4, MD-2, and LBP mRNA in the ileum of the GN minipigs were significantly induced in the presence of *S*. Typhimurium, but in these cases, the pig commensal bacteria were not changed compared to the GF control. In contrast, CD14 mRNA expression in the ileum was not related to the *Salmonella* presence. A possible explanation is that CD14 is not specific for the discrimination of LPS, but also recognizes other structures in relation to TLR2 [71,72]. These findings agree with our previous results in preterm minipigs colonized with probiotic *Lactobacillus rhamnosus* GG [62,73] and *B. animalis* subsp. *lactis* BB-12 [74,75]. A similar situation was found in the ileum of GN minipigs colonized with pig commensal lactobacilli *L. amylovorus* P1 and *L. mucosae* P5 but not with probiotic *E. coli* Nissle 1917 [76]. This probiotic EcN was effective in preventing infant and toddler acute diarrhea [77,78] and acute diarrhea in *S*. Typhimurium-infected GN minipigs [79]. One reason for the high effectivity of EcN could be its rough chemotype LPS (R-LPS) [80]. GN minipigs colonized with R-LPS *S*. Typhimurium F98 or *S*. Typhimurium SF1591 were resistant to subsequent infection with virulent *S*. Infantis 1326/28 [81,82] or *S*. Typhimurium LT2 [83], respectively. *S*. Typhimurium LT2 is avirulent for one-week-old CV piglets [53], which are protected by complex microbiota mediating their colonization resistance [26,27]. In contrast, *S*. Typhimurium LT2 is lethal for their GF counterparts with low colonization resistance [52]. Prepared Δ*rfa* LT2 mutants with R-LPS were less competent in triggering the TLR4-signaling and inducing inflammatory cytokines so that they did not exceed their beneficial regulatory range in the protective inflammatory reaction. In contrast, wild-type LT2 induced excessive levels of inflammatory cytokines that were detrimental to GN minipigs [52,84].

TLRs are multiligand receptors [5,7]. Thus, various ligands can influence their upregulation such as, e.g., HMGB1, which is the ligand of all observed TLRs (2, 4, and 9) in our experiments. HMGB1 can be released in tissue damage and can reflect the severity of sepsis [85], e.g., the destruction of ileal villi in *Salmonella*-infected minipigs [61,74], and released HMGB1 can emphasize an inflammatory reaction through its cytokine activity [86]. Moreover, an HMGB1-LPS complex can present LPS for TLR4/MD-2 recognition [87] and amplify an inflammatory reaction.

TLR2 recognizes Gram-positive bacterial structures in the heterodimers TLR2/TLR1 and TLR2/TLR6 [7]. TLR2 was not induced with pig commensal RP36, RP37, and LA compared to the GF minipigs, but its mRNA expression in the ileum was upregulated by *Salmonella* concordantly with TLR4. TLR2 can also participate in the recognition of Gram-negative structures. The TLR2/TLR1 heterodimer recognizes curli amyloid fibrils of Gram-negative *S*. Typhimurium biofilm [71]. This should explain the similar expression of TLR2 and TLR4 in the presence of *Salmonella*. In contrast to TLR2 and TLR4, TLR9 mRNA expression was not modulated in the presence of pig commensal bacteria or *S*. Typhimurium compared to the GF counterparts. Similarly to our results, TL2 and TLR4 mRNA expression in the ileum was upregulated in CV pigs 24 h after infection with *S*. Typhimurium, but TLR9 mRNA expression was not influenced [88].

Various TLRs use MyD88 or TRIF adaptor proteins. TLR2 and TLR9 use MyD88 adaptor protein for downstream signaling, but TLR4 can use both adaptor proteins in MyD88-dependent and TRIF-dependent pathways according to the type of ligand and localization of the TLR4 on the cellular membrane or in the endosome [88]. MyD88 mRNA expression showed a similar trend to TLR2 and TLR4 mRNA expression with upregulation in the presence of *Salmonella*, but TRIF mRNA did not show any apparent trend. Thus, we suppose the primary TLR4 signaling was through the MyD88-dependent pathway. The presence of bacteria did not influence RAGE mRNA expression. However, RAGE is a multiligand receptor capable of binding to a broad range of structurally diverse ligands expressed on various cell types and participating in various physiological functions and pathological processes. Moreover, cleaved RAGE can occur in two forms of soluble RAGE and can act as a decoy receptor and regulate downstream signaling [89].

The colon is the site of the densest bacterial population of the GIT [90,91], and *Salmonella* occurrence is abundant [57,68]. The *Salmonella* CFU density was found one order higher in the colon compared to the ileum in CV piglets at 2 days post-infection [92]. A similar colon/ileum CFU ratio was found in *Salmonella*-infected GN minipigs [61]. The *Salmonella* infection upregulated LBP, CD14, TLR4, and LBP in the colon. Both MyD88 and TRIF adaptor protein mRNA expression showed an apparent trend related to the infection. MyD88 was upregulated as it was in the ileum, but the TRIF mRNA expression was downregulated by the infection with *Salmonella*. This was also observed in the colon of the GN minipigs challenged/colonized with Δ*rfa S*. Typhimurium strain LT2 mutants, where TRIF mRNA expression increased with truncation of LPS [52]. We believe that the MyD88-dependent signaling pathway is the crucial pathway in the case of the ileum and in the case of the colon, but the downregulation of the TRIF-dependent pathway by *Salmonella* infection and its relation to the LPS chain needs future studies.

Experiments in vitro do not fully cover the complexity of microbiota-host interactions, but they bring valuable findings that can help elucidate microbiota-host and microbiota-microbiota interferences, including the role of TLRs in inflammatory signaling. Porcine epithelial cell line IPEC-J2 infected with *S*. Typhimurium showed upregulated TLR2 mRNA expression 1.5 and 6 h post-infection. However, no TLR4 and TLR9 mRNA expression changes were found within the observed 6 h period [88]. *L. amylovorus* DSM 16698^T^, which we used in our in vivo experiment, suppressed TLR4 signaling in human Caco-2/TC7 cells and pig jejunal explants, as well as the overproduction of inflammatory cytokines IL-8 and IL-1β, by inhibiting ETEC-induced TLR4 and MyD88 mRNA expression. This anti-inflammatory effect was achieved by modulating the negative regulators Tollip and IRAK-M. Anti-TLR2 antibodies proved the role of TLR2 in suppressing TLR4 signaling [93].

More than 200 biomarkers of sepsis are described, such as C-reactive protein (CRP), procalcitonin, calprotectin, and some inflammatory cytokines, e.g., tumor necrosis factor (TNF)-α and interleukin (IL)-6, IL-8, IL-10, and IL-12 [94,95]. Plasmatic cytokine levels help to discriminate between physiological (homeostasis) and pathological (inflammation) processes [19]. We selected two inflammatory markers—IL-6 and IL-12/23p40 to assess the activation of innate immune response and possible cytokine storm [95]. IL-6, IL-12, and IL-23 belong to the ‘level 1′ cytokines that stimulate the production of other cytokines through various cells [96].

IL-6 is a pleiotropic cytokine that participates in hematopoiesis and governs acute phase response [97]. Its detection is a helpful marker of neonatal sepsis [98]. In the intestine, enterocytes produce IL-6 with anti-inflammatory and cell-protective properties that strengthen the intestinal barrier and can alleviate its disruption by *Salmonella* infection [99]. GN minipigs challenged with necrotoxigenic *E. coli* O55 (EcO55) without significant signs of enteric infection showed low or undetectable levels of systemic IL-6, but minipigs with significantly expressed clinical signs of enteric infection showed high levels of IL-6 24 h post-infection [100]. In our experiments, none of the used pig commensal bacteria increased levels of IL-6 in plasma. The infection with LT2 significantly increased plasmatic IL-6 levels. The previous association with mucinolytic *B. boum* RP36 did not influence this increase. In contrast, the previous association with non-mucinolytic *B. boum* RP37 and *L. amylovorus* prevented IL-6 increase. Differences in the diminishing of plasmatic IL-6 induced by *B. boum* strains agree with previous findings in TNF-α and IL-10 decreasing, but in these cases, only non-significantly [57].

The inner lumen of the ileum (intestine in general) is covered with a mucin layer that prevents the tight contact of enterocytes with bacteria and their translocation. Moreover, the upper movable mucin layer helps to clear away bacteria from the intestine via peristalsis [101]. The disruption of the protective mucin layer by mucinolytic RP36 [57] could support *Salmonella* translocation, which could result in the upregulation of IL-6. The highest downregulating effect was shown for *L. amylovorus* DSM16698^T^. This *Lactobacillus* showed a suppressive effect on inflammatory cytokines in Caco-2/TC7 cell line and piglet explants infected with ETEC K88, as mentioned about TLR4 regulation [93].

IL-12 and IL-23 are critical cytokines for human immunity against *Salmonella* [102]. They are primarily produced by antigen-presenting cells (APCs) and regulate colonization resistance and intestinal inflammation [103]. IL-12 and IL-23 are heterodimeric pro-inflammatory cytokines composed of p40 and p35 or p40 and p19 units, respectively [104]. GN minipigs infected with EcO55 that suffered from infection had significantly increased intestinal IL-12/23p40 levels, which correlated with plasmatic levels of IL-12/23p40. The IL-12/23p40 levels were directly related to clinical signs of enteric infection and sepsis [100]. In the present work, we used IL-12/23p40 as a biomarker of enteric infection and sepsis and a marker of the possible effectivity of commensal pig bacteria (RP36, RP37, and LA) to modulate the TLR4/MD-2 signaling pathway. None of them prevented an increase in IL-12/23p40 levels after infection with *S*. Typhimurium.

## 5. Conclusions

Gnotobiotic animals allow the study of interferences between hosts and simple and defined microbiota. Obtained results can reflect the particular activity of individual bacteria but also can mirror artificial conditions that naturally do not occur. Concordantly, both variants can bring interesting information. More complex microbiota, e.g., multi-strain probiotic preparations, can reflect more complex actions compared to single-strain species [104,105]. Within the last decade, synthetic, defined multi-strain microbiota have been developed with more complex synergy and additive effects, as shown in experiments with gnotobiotic mice [105,106]. In relation to this trend, we plan to use defined multi-species microbiota composed of pig commensal bacteria with selected properties [107] to study more complex host-microbiota interferences under defined conditions.

## Figures and Tables

**Table 1:** Villus height, crypt depth, and ratio of villus height/crypt depth in the terminal ileum of the GN minipigs. These characteristics in the terminal ileum in the non-infected minipigs (GF, RP36, RP37, and LA) were evaluated using ANOVA with Tukey’s post-hoc test and presented as mean ± SD. The values from six minipigs per group were compared (*p* < 0.05).

	GF	RP36	RL37	LA
**Villus height** (μm)	704.9 ± 76.2	614.2 ± 71.4	614.8 ± 40.1	635.9 ± 56.5
**Crypt depth** (μm)	74.7 ± 3.8	77.2 ± 7.8	78.7±4.3	77.2 ± 6.1
**Height/Depth** (ratio)	9.1 ± 2.1	8.2 ± 1.6	6.9 ± 0.8	8.0 ± 1.3

## Data Availability

The corresponding author will share the data on request.
